# Genome-Wide Analysis of *Potassium Channel* Genes in Rice: Expression of the *OsAKT* and *OsKAT* Genes under Salt Stress

**DOI:** 10.3390/genes12050784

**Published:** 2021-05-20

**Authors:** Zahra Musavizadeh, Hamid Najafi-Zarrini, Seyed Kamal Kazemitabar, Seyed Hamidreza Hashemi, Sahar Faraji, Gianni Barcaccia, Parviz Heidari

**Affiliations:** 1 Department of Biochemical Sciences, Sapienza University of Rome, 00185 Rome, Italy; Zahra.musavizadeh@uniroma1.it; 2 Department of Plant Breeding, Sari Agricultural Sciences and Natural Resources University (SANRU), Sari 4818166996, Iran; najafi316@yahoo.com (H.N.-Z.); kazemi_ka@yahoo.com (S.K.K.); sahar.faraji@rocketmail.com (S.F.); 3 Genetics and Agricultural Biotechnology Institute of Tabarestan, Sari Agricultural Sciences and Natural Resources University, Sari 4818166996, Iran; irahamidreza@yahoo.com; 4 Laboratory of Genomics for Breeding, DAFNAE, Campus of Agripolis, University of Padova, Legnaro, 35020 Padova, Italy; gianni.barcaccia@unipd.it; 5 Faculty of Agriculture, Shahrood University of Technology, Shahrood 3619995161, Iran

**Keywords:** in silico study, potassium transporters, protein–protein interaction, comparative genomics, posttranslational modifications, gene expression

## Abstract

Potassium (K+), as a vital element, is involved in regulating important cellular processes such as enzyme activity, cell turgor, and nutrient movement in plant cells, which affects plant growth and production. Potassium channels are involved in the transport and release of potassium in plant cells. In the current study, three *OsKAT* genes and two *OsAKT* genes, along with 11 nonredundant putative *potassium channel* genes in the rice genome, were characterized based on their physiochemical properties, protein structure, evolution, duplication, in silico gene expression, and protein–protein interactions. In addition, the expression patterns of *OsAKTs* and *OsKATs* were studied in root and shoot tissues under salt stress using real-time PCR in three rice cultivars. *K+ channel* genes were found to have diverse functions and structures, and *OsKATs* showed high genetic divergence from other *K+ channel* genes. Furthermore, the Ka/Ks ratios of duplicated gene pairs from the *K+ channel* gene family in rice suggested that these genes underwent purifying selection. Among the studied K+ channel proteins, OsKAT1 and OsAKT1 were identified as proteins with high potential N-glycosylation and phosphorylation sites, and LEU, VAL, SER, PRO, HIS, GLY, LYS, TYR, CYC, and ARG amino acids were predicted as the binding residues in the ligand-binding sites of K+ channel proteins. Regarding the coexpression network and KEGG ontology results, several metabolic pathways, including sugar metabolism, purine metabolism, carbon metabolism, glycerophospholipid metabolism, monoterpenoid biosynthesis, and folate biosynthesis, were recognized in the coexpression network of K+ channel proteins. Based on the available RNA-seq data, the *K+ channel* genes showed differential expression levels in rice tissues in response to biotic and abiotic stresses. In addition, the real-time PCR results revealed that *OsAKTs* and *OsKATs* are induced by salt stress in root and shoot tissues of rice cultivars, and *OsKAT1* was identified as a key gene involved in the rice response to salt stress. In the present study, we found that the repression of *OsAKTs*, *OsKAT2*, and *OsKAT2* in roots was related to salinity tolerance in rice. Our findings provide valuable insights for further structural and functional assays of *K+ channel* genes in rice.

## 1. Introduction

Potassium (K+), as a fundamental macronutrient, is essential for plant growth and plays an important role in regulating cellular processes such as the control of pH and cell turgor [1,2]. Potassium is also involved in regulating the activity of many enzymes as an essential cofactor [3,4]. In addition, K+ is required for the transport of phloem solute and maintaining the balance of cations:anions in the cytosol and the vacuole [5]. Furthermore, potassium plays a vital role in plant adaptation to abiotic stresses, including salinity and drought [6,7], and biotic stresses, including pathogen attack and the resulting wounds [8,9]. Changing the concentration of potassium in plant tissues and cells effectively regulates the response of plants to environmental changes [4]. For example, to counteract the harmful effects of other ions, the potassium concentration in the cytoplasm is in the range of 100–200 mM and is not replaced by other cations, while the potassium concentration in the vacuoles is variable and can be replaced by other osmotica [10,11]. This replacement is effective in maintaining cellular pressure. Potassium is transported by various systems in plant cells. K+ channels are involved in the transport and release of potassium [1,12]. K+ channels are grouped into different categories according to the type of activity, influx, and efflux [2,13]. K+ channels are found in cell membranes, vacuolar membranes, xylem, and phloem tissues that participate in maintaining homeostasis and the transport of potassium within the plant cell [1,12,13]. K+ channels are involved in K+ loading in xylem and phloem, as well as accumulation in vacuoles [13]. A broad range of genes encoding K+ channels have expanded during evolution and obtained specific functions in plants [12]. Many K+ channel proteins linked to the uptake and release of K+ from the cell have been stated in different plant species [2,14,15].

K+ channels are divided into two main categories, including voltage-gated K+ channels and voltage-independent K+ channels, based on their mechanisms [3,16]. In plants, four subgroups of voltage-gated plant K+ channels have been segregated, including Outward-rectifying K+ (K_out_) channels, Inward-rectifying K+ (K_in_) channels, Weak-rectifying K+ (K_weak_) channels, and Silent-rectifying K+ (K_silent_**)** [17]. Besides, various *K+ channel* genes have been identified and characterized in plant species. For instance, in Arabidopsis, *KAT1* and *AKT1* were identified as inward-rectifying shaker-like K+ channels (K_in_), in which *KAT1* is involved in the transfer of potassium in guard cells [18,19,20]. *AKT1*, a significant factor in the uptake of K+, is highly expressed in root epidermal cells [16,21,22], while *AKT2* is identified as a weak-rectifying (K_weak_) channel [3]. Furthermore, the Guard cell Outward-Rectifying K+ (GORK) channel, as a K+ efflux channel (K_out_) in guard cells, induces stomatal closure [23,24]. The rice genome contains three *OsKAT* genes, *OsKAT1*, *OsKAT2*, and *OsKAT3*, which encode inward rectifying shaker-like potassium channels, and two *OsAKTs* (*OsAKT1* and *OsAKT2*) [25,26]. Moon et al. found that *OsKAT2*, a rice-*KAT* gene, is mainly expressed in guard cells involved in stomatal opening and was introduced as part of the response to drought stress [25]. It has also been stated that the *OsKAT1* gene increases salinity tolerance in rice [26]. One of the critical aspects of the effect of potassium in plant cells is the interaction of this element with sodium (Na+), which is useful in increasing plant tolerance to salinity stress [27]. The activity of K+ channels is affected by salinity. For instance, *OsAKT1* transcription is downregulated in response to salt stress [28], while the expression levels of *AKT2/3*, as a gene localized in the phloem, are upregulated by salinity [29]. *AKT2/3* is probably involved in controlling K+ homeostasis in plants and the K+/Na+ ratio by controlling the recirculation of K+ in the phloem [30]. It has also been stated that posttranslational modifications such as phosphorylation can affect the AKT1 protein and increase the channel activity of AKT1 [21,31].

A comprehensive genomic study of potassium channel proteins can reveal valuable information on the sequence structure and interaction between these proteins. To date, studies have been conducted on the function of these genes in different plants, but many regulatory and structural aspects of these proteins are still unknown. In the current study, potassium channel proteins, especially OsAKTs and OsKATs, were compared based on their sequence structure, physicochemical properties, protein interaction network, posttranslational modifications, and expression profile in rice. Overall, the reported results in the present study will enhance our knowledge about the evolution and function of *OsAKTs* and *OsKATs* and can serve as a basis for revealing the regulatory mechanism and functional genomic analysis of *AKT* and *KAT* genes.

## 2. Materials and Methods

### 2.1. Identification of Potassium Channel Proteins in Rice

The HMM profile related to the K+ channel protein domains (PF07885, PF00520, PF11834) was first retrieved through the Pfam database [32], and an HMM search (HMMER3.0) was conducted to detect the putative K+ channel proteins in the rice genome (*Oryza sativa* Japonica Group cv. Nipponbare), with an expected value of E^−10^. The recognized nonredundant putative K+ channel proteins were manually checked for specific domains by employing the Pfam and SMART [33] programs. The corresponding cDNA and genomic sequences of the identified proteins, as well as the chromosomal location of *K+ channel* genes, were obtained from the EnsemblPlants database [34]. The physicochemical properties of K+ channel proteins, such as molecular weights and isoelectric points (*pIs*), were identified through the ProtParam program [35].

### 2.2. Phylogenetic Relationships, Conserved Protein Motifs, and Gene Structures

K+ channels protein sequences from rice were used as the queries, to identify their orthologous in *Arabidopsis thaliana*, *Brachypodium distachyon*, *Zea mays*, and *Sorghum bicolor* using the BLAST tool of the EnsemblPlants database. The phylogenetic relationships were investigated by constructing a neighbor-joining phylogenetic tree via MEGAX software [36] according to the protein sequences of K+ channels from rice, *A. thaliana*, *B. distachyon*, *Z. mays*, and *S. bicolor* with 1000 bootstrap replicates. The MEME (Multiple Em for Motif Elicitation) server was also employed to discover the conserved protein motifs in K+ channel proteins [37]. The exon/intron organizations in the genes were predicted through the Gene Structure Display Server [38].

### 2.3. Chromosomal Mapping, Gene Duplications, and Estimation of the Ka/Ks Ratio

The *K+ channel* genes were mapped onto the rice chromosomes using MapChart software [39]. The duplication events were identified by alignment of the coding DNA sequences of *K+ channel* genes via CLUSTALW [40], and then the matrix was imported into BioEdit software (v. 7.2.5) [41]. Gene duplication was determined as genes sharing more than 80% identity in their nucleotide sequences. The synonymous (Ks) and nonsynonymous (Ka) rates per site among the duplicated pairs were calculated using DnaSP v6 software [42]. The time of division of duplicated pairs was estimated using a synonymous mutation rate of λ substitutions per synonymous site per year, as T = (Ks/2λ (λ = 6.5 × 10^−9^)) × 10^−6^ [43]. The synteny relationships at both the gene and chromosome levels of *K+ channel* genes in rice with their orthologous pairs in maize, Arabidopsis, *B. distachyon*, and *S. bicolor* were visualized by Circos software [44].

### 2.4. Coexpression Network of K+ Channel Genes

The co-expression networks related to *K+ channel* genes were constructed using the ATTED-II ver 9.0 server [45] with the coex option on many genes and PPI option on a few genes; finally, the results were visualized with Cytoscape [46]. The genes directly connected with K+ channel proteins in various biological pathways were further investigated based on RNA-seq and KEGG (Kyoto Encyclopedia of Genes and Genomes) data on the gene networks.

### 2.5. 3D Protein Modeling, Validation, and Docking Analysis of the Pocket Sites

The three-dimensional structures of the K+ channel proteins in rice were predicted through the Protein Homology/analogy Recognition Engine V 2.0 (Phyre2) server [47]. The predicted protein model validation was assessed through Ramachandran plot analysis [48]. Docking analysis of the ligand-binding regions in the predicted protein models was also performed via CASTp [49] and DeepSite [50] tools and finally visualized in PyMOL [51].

### 2.6. Expression Profiling of K+ Channel Genes Based on RNA-Seq Data

The available RNA-seq data related to the rice genome were employed for expression assays of K+ channel family members in multiple tissues and during exposure to various biotic and abiotic stimuli. These data contained a wide range of developmental stages of rice, including leaves at 20 days, pre- and postemergence inflorescences, anthers, pistils, seeds, embryos, endosperm, seedlings, calli, and panicles. In addition, the gene expression data under different stresses, including phosphate starvation (Pi), cadmium (Cd), drought (DR), salinity (S), and arsenic (As) stresses, were downloaded from the Rice Expression Database [52] (SRP028766, DRP001141, SRP052306, DRP002329, SRP011893) [53,54,55]. In addition, the RNA-seq data of rice under mock, blast infection, *Xanthomonas oryzae*, and bacterial blight disease were downloaded from public data [56,57]. The transcript magnitudes were determined in fragments per kilobase per million mapped reads (FPKM) based on the exon model and then log2 transformed to generate heatmaps via the TBtools package [58].

### 2.7. Plant Materials and Stress Treatments

The seeds of three rice cultivars, IR29, Sang tarom, and Jelodar, were collected from the Mazandaran Province in Iran. The studied cultivars differed in their salinity tolerance rate: Sang tarom was a moderately tolerant cultivar; IR29 was a susceptible cultivar; and Jelodar was a moderately susceptible cultivar. Seeds were sterilized in 0.2% (*w*/*v*) sodium hypochlorite for 1 min and then placed in petri dishes containing two layers of Whatman filter paper. All petri dishes were incubated at a temperature of 25 ± 3 °C. After four days, germinated seeds were moved to a Yoshida solution [59] at pH 5–5.5 for further growth. In the present study, we used aerated hydroponic tanks holding 30 L solution, and 80 seedlings were vertically cultured in each tank. The nutrient solution was exchanged once a week, and the pH was adjusted every three days. Seedlings were grown at 25 ± 3 °C, a 16 h photoperiod, and 60–65% relative humidity. Three-week-old seedlings were subjected to 120 mM NaCl treatment. Salt-treated plants were harvested after 6 h, 24 h, 72 h, 120 h, and 168 h time courses. Nontreated seedlings were also considered the control sample. Collected root and leaf samples were frozen immediately in liquid nitrogen and stored at −80 °C until use.

### 2.8. RNA Isolation and Quantitative Real-Time PCR Analysis

Total RNA was extracted using TRIzol reagent (Invitrogen, Carlsbad, CA, USA) according to the manufacturer’s instructions. The purified total RNA was checked using agarose gel electrophoresis and a NanoDrop ND 1000 Spectrophotometer (Wilmington, DE, USA). *DNase I* treatment was performed to eliminate genomic DNA contamination using a ThermoFisher (Thermo Fisher Scientific, Wilmington, MA, USA) DNase Kit according to the manufacturer’s instructions. *DNase I*-treated RNA was used to synthesize first-strand cDNA using the QuantiTect Reverse Transcription Kit (Qiagen, Hilden, Germany) and oligo (dT) primers in a 20 μL final volume, according to the manufacturer’s instructions. RT-PCR was performed using 3 μL RNA, 1 μL of specific primers (forward and reverse), 10 μM dNTPs, 5 × PCR buffer, and 1 μL RiboLock RNase Inhibitor. Finally, PCR was performed, and the products were separated on a 1% agarose gel to check quality the gene fragments. First-strand cDNA mixtures were utilized as templates for real-time PCR analysis.

The cDNA sequences of the *OsAKTs* and *OsKATs* in rice, including *OsAKT1* (*Os01g0648000*), *OsAKT2* (*Os05g0428700*), *OSKAT1* (*Os02g0245800*), *OsKAT2* (*Os01g0210700*), and *OsKAT3* (*Os01g0756700*), were selected for the transcription assay. The gene-specific primers were designed for candidate genes and internal controls using Primer3 online software for candidate genes and internal controls (Table S1). In the present study, the *β-actin* gene was used as an internal control for data normalization. Quantitative real-time PCR (qPCR) was performed in a 10 μL volume containing 2 μL of cDNA, 5 μL of 2 × SYBR Green Master Mix, 0.3 μL of each 10 μM primer, and 2.7 μL of RNase-free water. The amplification reactions were carried out in a two-step thermal cycler protocol (Thermo Scientific) according to the company’s procedures: a 10 min initial activation step at 95 °C, followed by 40 cycles of 95 °C for 15 sec and 60 °C for 1 min. After 40 cycles, the amplification specificity was checked based on the melting curves by heating the amplicons from 55 to 95 °C. In this study, each treatment of the experiment was repeated in three biological replicates. To increase the reliability of the gene expression analysis, real-time PCR experiments were performed with three identical technical replications. For quantitative real-time PCR data, the relative expression of genes was calculated based on the threshold cycle (CT) method. Accordingly, the relative expression level of the target genes was calculated by the 2^−∆∆CT^ equation [60]. A student’s t-test was applied to determine the significant difference (*p*-value < 0.05 and < 0.01) between the applied treatments (salt stress at different time courses) and control. In the current study, control samples included conditions without salt stress. All expression results were constructed using Prism 6 software (GraphPad Software Inc., San Diego, CA, USA) based on the mean and standard division (SD) of each gene.

## 3. Results

### 3.1. Genome-Wide Characterization of K+ Channel Genes

In the current study, 16 nonredundant putative *K+ channel* genes in the rice genome were identified and characterized based on their physicochemical properties and sequences (Table 1). The identified genes showed diverse functions in the inward and outward transport of potassium. Furthermore, the *K+ channel* genes encoded proteins with lengths from 286 (Os04g0117500; as a voltage-dependent K+ channel) to 935 (Os01g0648000; OsAKT1) amino acid residues and with an MW in the range between 33.54 (Os01g0696100; as an outward K+ channel) and 104.46 kDa (Os01g0648000; OsAKT1). In addition, the exon number varied from one (Os06g0254200; NKT5) to 11 (Os01g0648000, Os01g0210700, Os02g0245800, and Os06g0250600), and the pI ranged from 5.78 (Os06g0250600; as an outward shaker K+ channel) to 10.39 (Os04g0117500).

**Figure 1:**
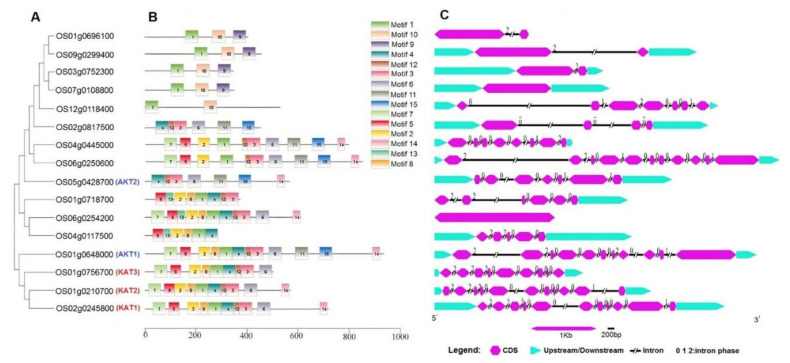
Phylogenetic analyses of the motifs in proteins and gene structures of *K+ channel* genes in rice. Phylogeny of K+ channel proteins (**A**), conserved motif distribution of K+ channel proteins (**B**), and intron-exon distribution of *K+ channel* genes (**C**).

According to the gene structure and conserved motif distribution, the *K+ channel* genes showed diverse sequence structures (Figure 1, panel A). In the present study, 15 conserved motifs were detected in K+ channel proteins, and motif 1 was observed in most proteins (Table S2). Three OsKAT proteins and two OsAKT proteins showed a similar motif distribution pattern (Figure 1, panel B). In addition, *K+ channel* genes were diverse in terms of gene structure, where various intron/exon numbers were observed (Figure 1, panel C).

### 3.2. Phylogenetic Analysis

**Figure 2:**
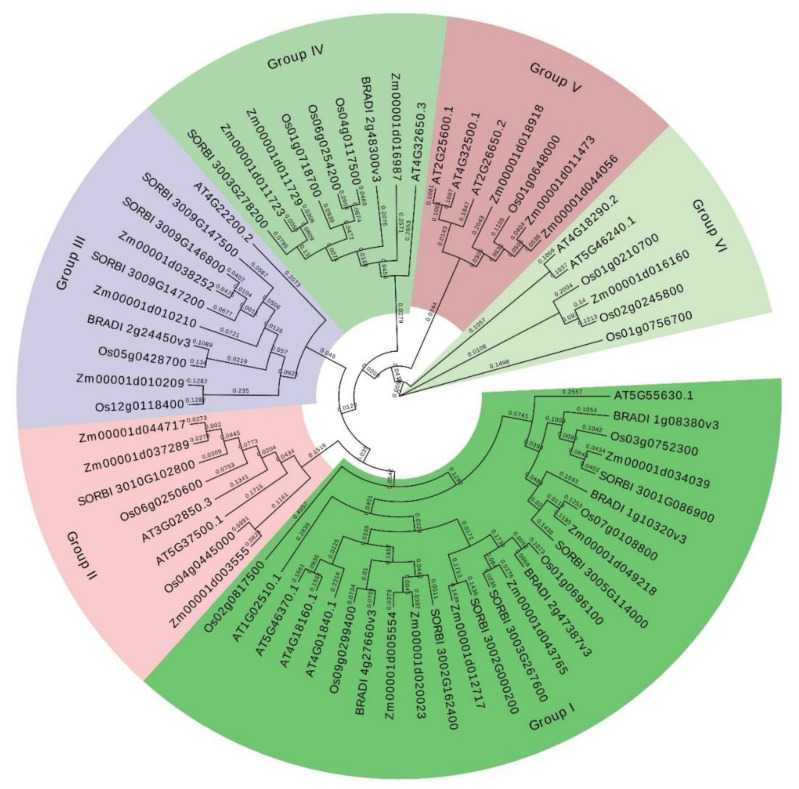
The phylogenetic tree of K+ channel proteins from rice along with protein sequences from four other species. The start of each gene ID contains the code for the species as Os: *O. sativa*; Zm: *Z. mays*; AT: *A. thaliana*; BRADI: *B. distachyon*; and SORBI: *S. bicolor*.

In the present study, the evolutionary relationships of 16 K+ channel proteins in rice with their orthologs in *A. thaliana*, *B. distachyon*, *Z. mays*, and *S. bicolor* revealed that K+ channel proteins could be divided into six groups (Figure 2). Group I, with 25 K+ channel proteins, was found to be the largest clade. Groups II, III, IV, V, and VI included 8, 10, 9, 7, and 6 K+ channel proteins, respectively. In addition, the members of group VI that contained the OsKATs showed high genetic divergence from other K+ channel proteins. The phylogenetic results illustrated that the K+ channel proteins of rice were most similar to their orthologous genes in maize. Moreover, *OsKAT3* (*Os01g0756700*) showed the greatest genetic divergence from other orthologous genes.

### 3.3. Genomic Distribution and Duplication Assay of K+ Channel Family Genes

**Figure 3:**
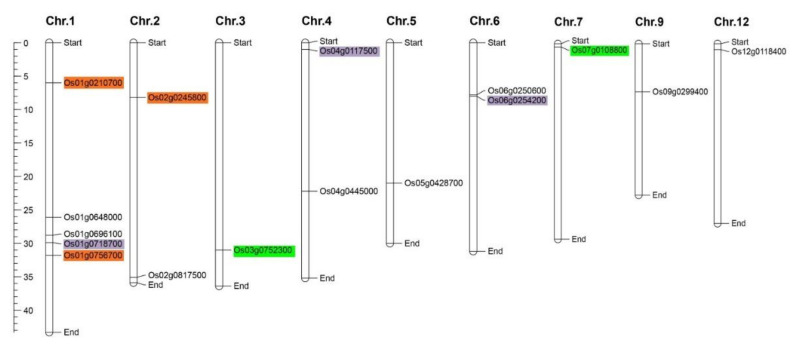
Location of *K+ channel* genes on the rice chromosomes. The pairs of segmentally duplicated genes are shown with the same color.

The *K+ channel* genes were mapped onto 9 out of 12 chromosomes in the rice genome. The chromosomal map illustrated an unequal distribution of the gene family members throughout the chromosomes (Figure 3). Chromosome 1 contained the largest number of *K+ channel* genes with five genes, while only one *K+ channel* gene was localized on chromosomes 3, 5, 7, 9, and 12. It was reported that many *K+ channel* genes in some plant species might be generated through gene duplication events, revealing a paleopolyploid origin for these important nutritional crops. Five segmental-duplicated gene pairs, categorized into three groups (including duplication and triplication events), were recognized in the *K+ channel* gene family. Each group has been denoted by different colors, revealing paralogous pairs (Figure 3). The highest numbers of duplicated/triplicated genes were distributed on chromosome 1, with three duplicated genes clustered into the various gene groups (Table S3). The intraspecies synteny results showed that some of the duplicated blocks were collinear, such as *Os01g0210700*, *Os01g0718700*, and *Os01g0756700* (Figure S1). The Ka/Ks magnitudes related to the paralogous pairs covered a domain from 0.105 to 0.236, and according to these ratios, duplication events were estimated to occur between approximately 84 and 204 million years ago (MYA) (Table S3). In addition, the Ka/Ks ratios were less than 1 in duplicated gene pairs from the *K+ channel* gene family in rice. This suggests that these genes have undergone purifying selection after duplication [61,62].

### 3.4. Conserved Synteny Relationships of K+ Channel Genes

**Figure 4:**
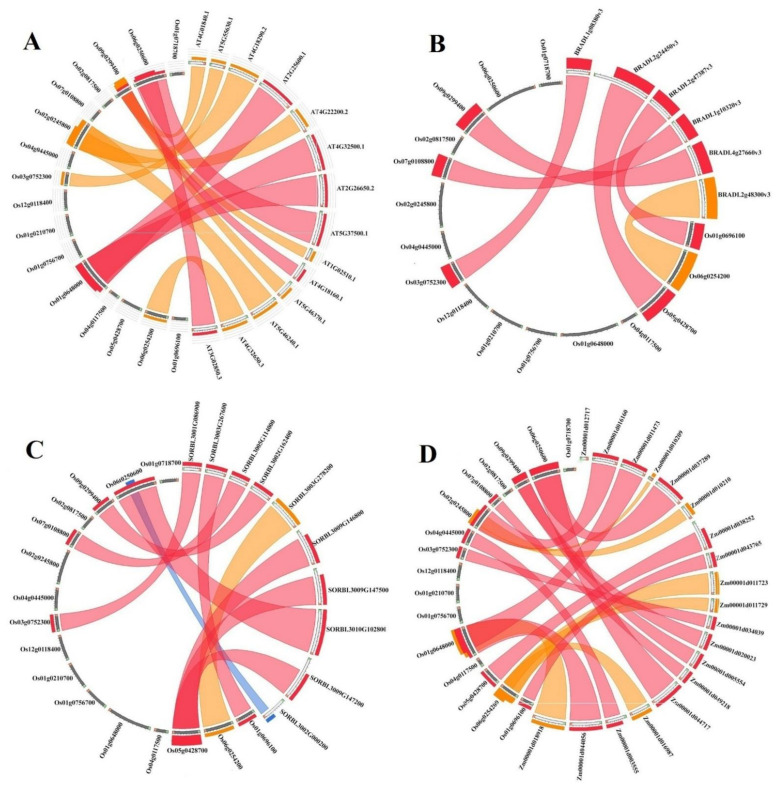
Synteny analysis of *K+ channel* genes. The syntenic blocks of rice *K+ channel* genes were compared with their orthologs in *A. thaliana* (**A**), *B. distachyon* (**B**), *S. bicolor* (**C**), and *Z. mays* (**D**).

To further study the evolutionary processes of *K+ channel* genes, the synteny analysis was performed between *K+ channel* genes from rice and their orthologs in *A. thaliana*, *B. distachyon*, *S. bicolor*, and *Z. mays* (Figure 4). As to the results, 14 syntenic blocks of orthologs were predicted between *O. sativa* and *A. thaliana K+ channel* genes (Figure 4A). Besides, *OsAKT1* showed syntenic regions with three orthologs in Arabidopsis, including *AtAKT1* (*AT2G26650*), *AtAKT5* (*AT4G32500*), and *AtAKT6* (*AT2G25600*), while *OsKAT1* had synteny with three *KATs* from Arabidopsis, including *AtKAT1* (*AT5G46240*), *AtKAT2* (*AT4G18290*), and *AtAKT2* (*AT4G22200*). The *K+ channel* genes in rice also showed an important syntenic relationship with their orthologs in the *B. distachyon* and *S. bicolor* genomes, with 6 and 10 syntenic regions, respectively (Figure 4B,C). *OsAKT2* showed synteny with three *K+ channel* genes in *S. bicolor*. In this study, 18 syntenic blocks were predicted between *K+ channel* genes from rice and their orthologs in maize (Figure 4D). Overall, the most segmental duplications were predicted between K+ channel orthologs in rice and maize. In the current study, *Os09g02994* and *Os03g0752300* were found in all syntenic blocks of orthologs, indicating that they are the most conserved genes in *K+ channel* gene family.

### 3.5. 3D Protein Modeling and Docking Analysis of Ligand-Binding Regions

**Figure 5:**
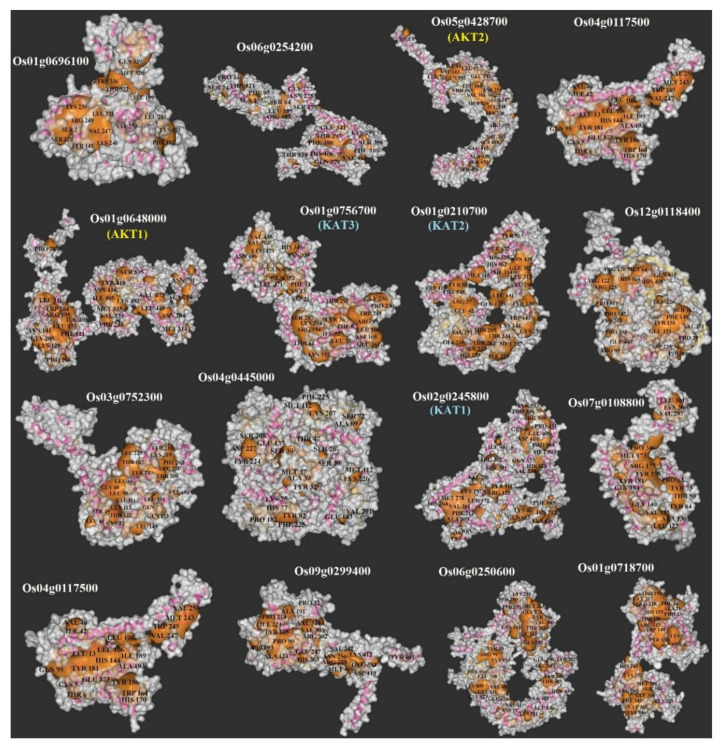
Docking analysis of pocket sites of K+ channel proteins in rice.

**Figure 6:**
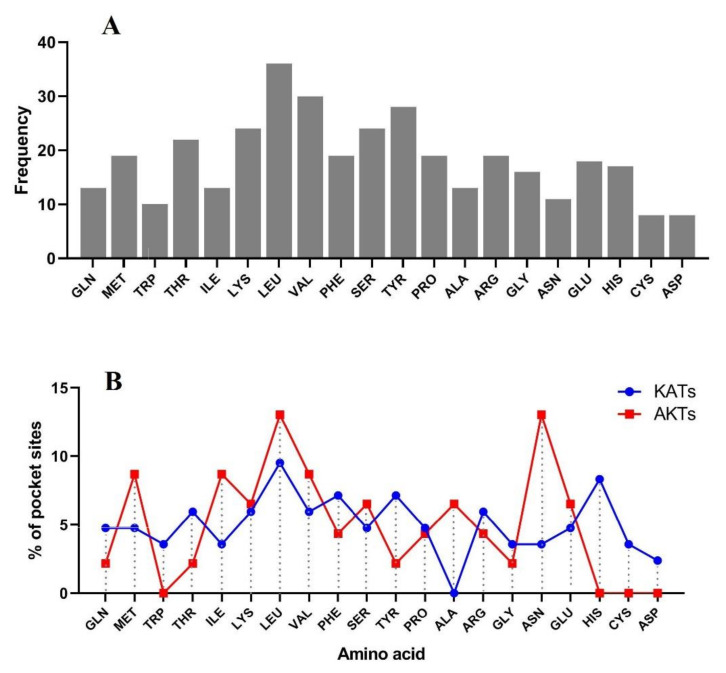
Distribution of ligand-binding sites in all studied K+ channel proteins (**A**), and the percentage of each ligand-binding site in predicted pocket sites of the KATs and AKTs subfamily in rice (**B**).

The protein structures of all the candidate K+ channel proteins were modeled at >90% confidence, and their potential active ligand-binding sites were also identified. According to the protein structure results, different active ligand-binding sites were predicted to be K+ channel proteins (Figure S2). Some diversity in the protein structure may reflect their different roles in the transmembrane transport process in response to multiple environments. In addition, the binding region/active sites of K+ channel proteins were predicted. Based on the results, different pockets were observed, and the key amino acids involved in the function of K+ channel proteins were predicted (Figure 5). The LEU, VAL, TYR, SER, LYS, and THR amino acids were more predicted to be the binding residues in the ligand-binding site of nearly all candidate K+ channel proteins (Figure 6A). The frequency of amino acid residues present in each pocket site was different between OsAKTs and OsKATs (Figure 6B). In OsAKTs, ASN and LEU were more frequently observed, while LEU and HIS were more frequently observed in pocket sites of OsKATs. Overall, LEU is recognized as the key residue in predicted pocket sites in K+ channel proteins. In addition, OsKATs and OsAKTs are different based on pocket sites affecting on their functions. These results suggest the importance of these residues in these positions on the DNA molecule and, finally, the cellular functional performance.

### 3.6. Prediction of Posttranslational Modifications

**Figure 7:**
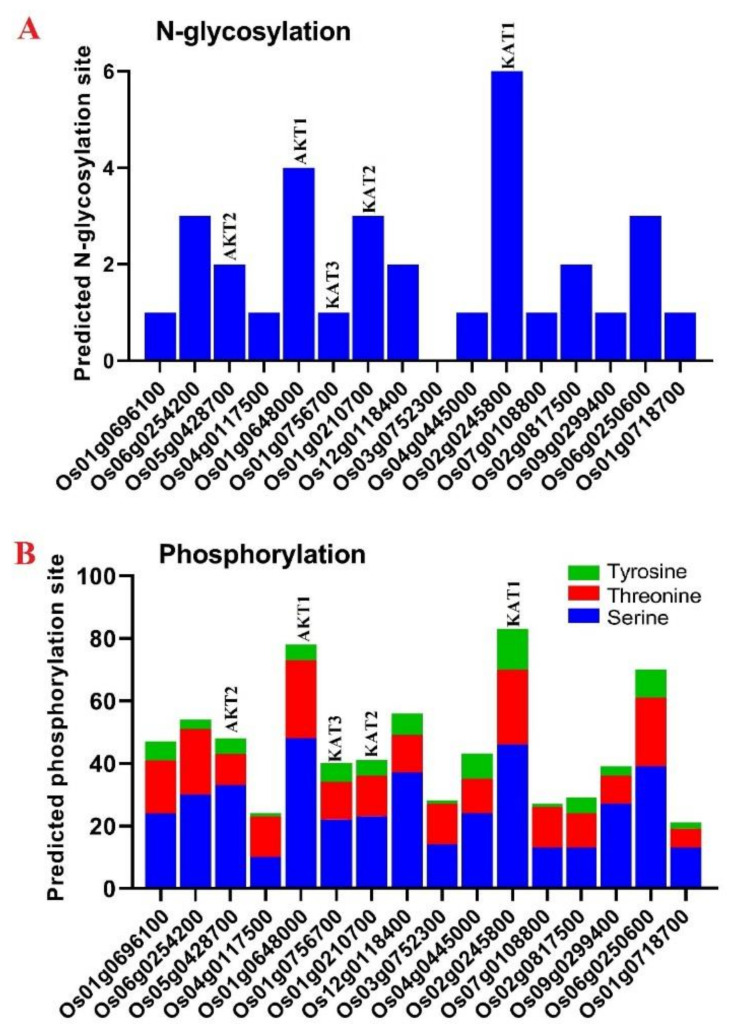
Prediction of posttranslational modifications in the amino acid sequences of K+ channel proteins of rice in terms of the N-glycosylation site (**A**) and phosphorylation site (**B**).

The potential N-glycosylation sites of potassium channel proteins were predicted, as shown in Figure 7. Except for Os03g0752300, OsKTPs showed potential N-glycosylation at one to six sites. As for the N-glycosylation results, OsKAT1 was predicted to be a protein with many glycosylation sites (six sites), while one glycosylation site was predicted in OsKAT3, and three sites were predicted in OsKAT2 (Figure 7A). In addition, four N-glycosylation sites were observed in OsAKT1, and two sites were observed in OsAKT2. Regarding the results of predicted phosphorylation sites, potassium channel proteins showed a range from 21 (in Os01g0718700) to 83 sites (in OsKAT1) (Figure 7B). Furthermore, 41 and 40 sites were predicted in OsKAT2 and OsKAT3, respectively (Figure 7B). In AKTs, 48 sites in OsAKT2 and 78 sites in OsAKT1 were predicted.

### 3.7. Expression Patterns of K+ Channel Genes in Different Tissues, Organs, and Stresses

**Figure 8:**
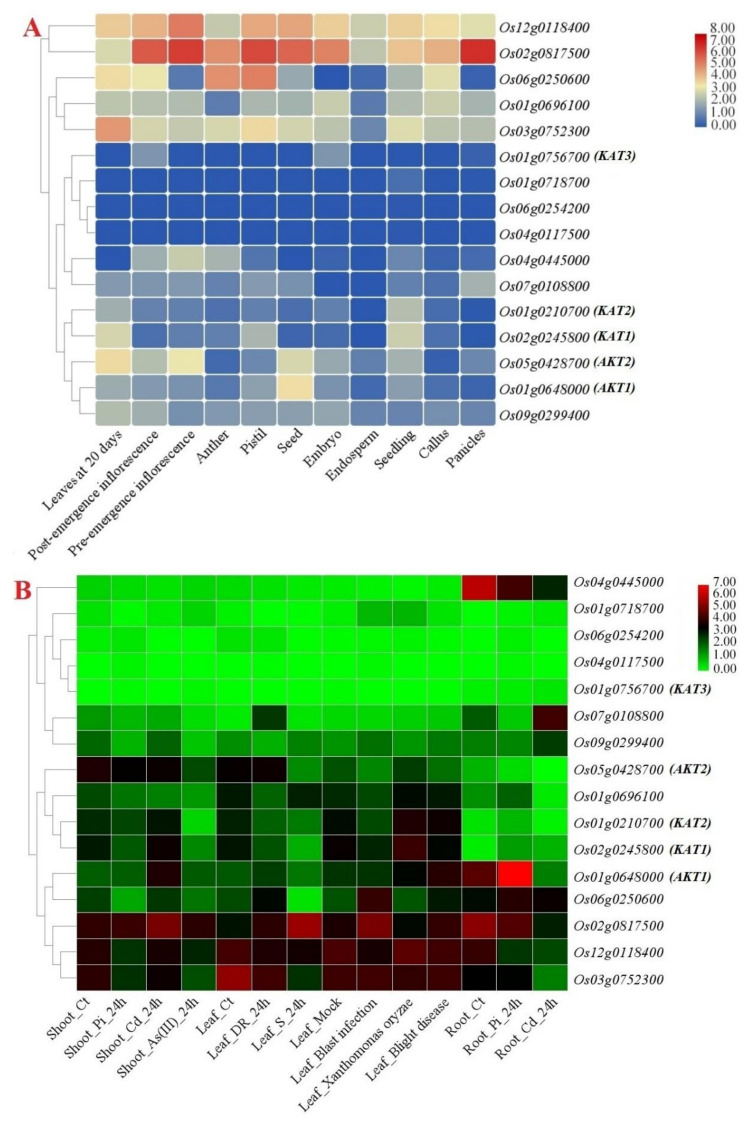
Expression heatmaps of *K+ channel* genes in rice from different tissues (**A**) and in response to abiotic and biotic stresses (**B**). The studied conditions included control (Ct); phosphate starvation (Pi), cadmium (Cd), drought (DR), salinity (S), and arsenic (As) stresses; and pathogens. Heatmaps were generated based on the log-2-transformed RNA-seq fragments per kilobase per million fragments mapped (FPKM) magnitudes in the exons of the rice genome.

The expression levels of potassium channel genes in different tissues, as well as in response to environmental stresses (biotic and abiotic), were examined using available RNA-seq data (Figure 8). The *K+ channel* genes showed differential expression and tissue-specific expression patterns (Figure 8A). *Os02g0817500*, a voltage-dependent potassium channel, showed high expression in all rice tissues, especially in panicles, pistils, and pre-emergence inflorescences (Figure 8A). In addition, *OsAKT1* was expressed in seeds and roots (Figure 8A and 8B), while *OsAKT2* was more highly expressed in leaves. Furthermore, the in silico expression profile of *K+ channel* genes was investigated under different stresses, including phosphate starvation (Pi), cadmium (Cd), drought (DR), salinity (S), and arsenic (As) stresses (Figure 8B). *K+ channel* genes showed differential expression in response to adverse conditions. For instance, *OsAKT1* was upregulated in response to phosphate starvation (Pi) in roots after 24 h, while *OsKAT1* was more induced in response to the rice pathogen *X. oryzae* in leaves (Figure 8B). In addition, *OsKAT3* was not expressed under stress and showed high divergence from other *OsKAT* genes based on the expression profile. As to the RNA-seq data related to stress conditions, *Os02g0817500* was detected as the *K+ channel* gene induced under all stimuli, while *Os12g0118400* and *Os03g0752300* were induced at a higher level in response to biotic stresses, indicating their important potential in stress resistance in rice (Figure 8B).

### 3.8. Coexpression Network Analysis of K+ Channel Genes

**Figure 9:**
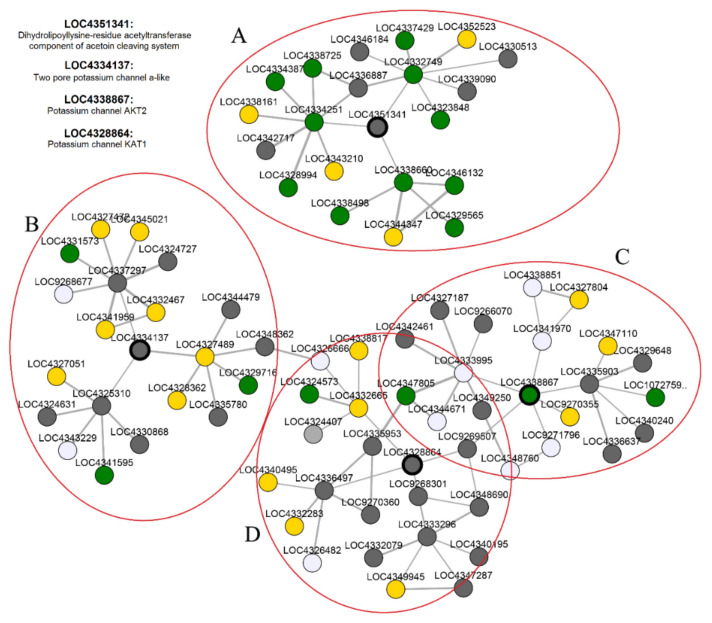
The co-expression network of *K+ channel* genes with other genes in rice. Ninety eight co-expressed genes clustered into the four co-expression nodes (**A**–**D**).

To obtain more insights regarding the *K+ channel* gene interactions with other genes in rice and their functional roles in plant cells, a co-expression network was constructed. As a result, 98 genes, clustered into the four co-expression nodes (A–D), were found in the co-expression network of *K+ channel* genes (Figure 9). According to the KEGG ontology results, the co-expressed genes were found to be involved in amino sugar and nucleotide sugar metabolism (KEGG ID: Osa00520), purine metabolism (KEGG ID: Osa00230), carbon metabolism (KEGG ID: Osa01200), glycerophospholipid metabolism (KEGG ID: Osa00564), monoterpenoid biosynthesis (KEGG ID: Osa00902), fructose and mannose metabolism (KEGG ID: Osa00051), and folate biosynthesis (KEGG ID: Osa00790). Among the *K+ channel* genes, LOC4338660 (UDP-sulfoquinovose synthase) in node A and LOC4327472 (probable mannose-1-phosphate guanylyltransferase 2) and LOC4345021 (probable mannose-1-phosphate guanylyltransferase 3) in node B, as neighbors of the *K+ channel* genes LOC4351341 and LOC4334137, were involved in amino sugar and nucleotide sugar metabolism. LOC4333995 (lecithin-cholesterol acyltransferase-like 1) in node C and LOC4325310 (phosphatide cytidylyltransferase 1) in node B were identified as co-expressed genes involved in glycerophospholipid metabolism. In node D, LOC4336497 (salutaridine reductase) and LOC4348690 (probable cinnamyl alcohol dehydrogenase 3) were identified as co-expressed with the *K+ channel* gene LOC4328864 in monoterpenoid biosynthesis. In node C, two genes, LOC4326296 (light-mediated development protein DET1) and LOC4352424 (E3 ubiquitin-protein ligase MIEL1), co-expressed with the *K+ channel* gene LOC4338867 (OsAKT2), were predicted to be engaged in ubiquitin-mediated proteolysis (Osa04120). LOC4328912 (cyclic nucleotide-gated ion channel 1) and LOC9266860 (probable calcium-binding protein CML18) were co-expressed with the *K+ channel* genes LOC4338867 and LOC4334137 in nodes C and B, respectively, and were predicted to be involved in the regulation of plant-pathogen interactions (Osa04626). Furthermore, LOC4336102 (indole-3-glycerol phosphate synthase) and LOC4327301 (ATP-dependent 6-phosphofructokinase 6) were found to be co-expressed with the *K+ channel* genes LOC4351341 and LOC4334137 during the biosynthesis of amino acids (Osa01230). The co-expression results revealed that *K+ channel* genes have critical functions during rice growth and responding to stimuli.

### 3.9. Expression of OsAKTs and OsKATs in Response to Salt Stress

**Figure 10:**
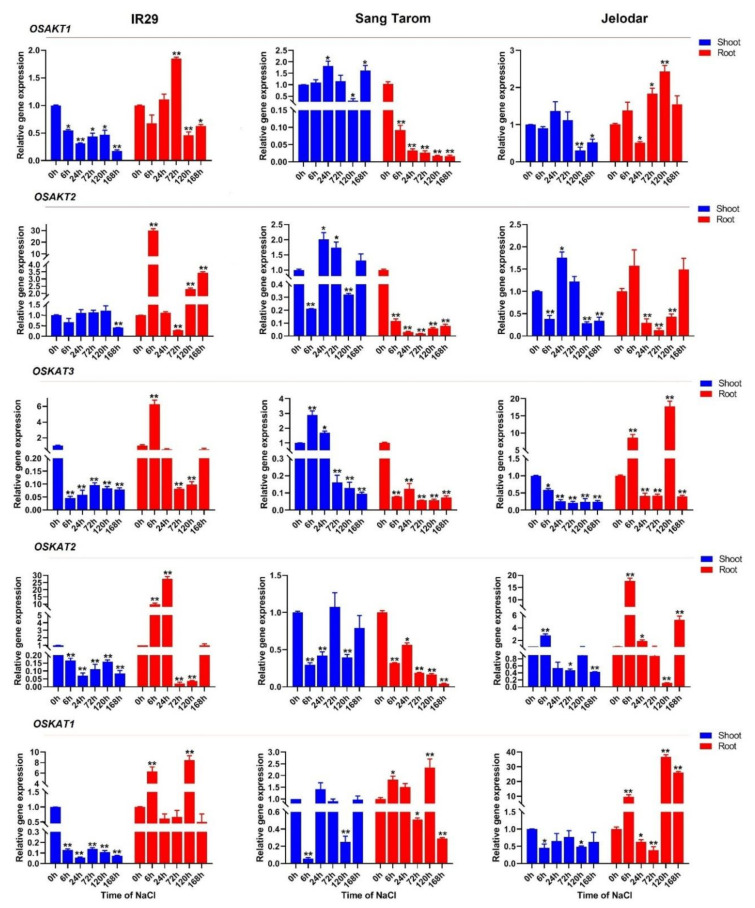
Relative expression levels of *OsAKT* genes (*OsAKT1* and *OsAKT2*) and *OsKAT* genes (*OsKAT1*, *OsKAT2*, and *OsKAT3*) in the shoot and root tissues of three rice cultivars, IR29 (susceptible cultivar), Sang Tarom (moderately tolerant cultivar), and Jelodar (moderately susceptible cultivar), in response to NaCl stress at different time courses. The values are given as the means ± SDs of three biological replicates, and the * and ** above the bars show a significant difference (according to Student’s *t*-test) between the applied treatments and the control at *p* < 0.05 and *p* < 0.01, respectively.

In the current study, *OsAKT* genes (*OsAKT1* and *OsAKT2*) and *OsKAT* genes (*OsKAT1*, *OsKAT2*, and *OsKAT3*) were selected for further assays of their potential in the response to salt stress in root and shoot tissues of three rice cultivars. The *OsAKTs* and *OsKATs* showed specific tissue expression, although this expression was somewhat dependent on the type of rice cultivar. For instance, *OsAKT1*, *OsAKT2*, and *OsKAT3* were more induced in shoot tissues of Sang Tarom, a tolerant cultivar, while all *OsAKTs* and *OsKATs* were upregulated in root tissues of the other two susceptible cultivars, IR29 and Jelodar, in response to salt stress (Figure 10). *OsAKT1* showed an upregulation in roots after 72 h of salt stress in IR29 and 72 to 120 h after salt stress in the Jelodar cultivar, while it was highly downregulated after 168 h in shoot tissues in both cultivars, IR29 and Jelodar. However, *OsAKT1* was downregulated at all time points of salt stress in root tissues of the Sang Tarom cultivar. *OsAKT1* showed high expression in the early stage of salinity stress (6 h) in root tissues of IR29, and it was highly downregulated after 72 h in root tissues in all studied rice cultivars (Figure 10). Based on the real-time PCR results, *OsKAT* genes showed higher expression in root tissues compared to other tissues in response to salt stress. *OsKAT3* showed a high expression in root tissues of the Jelodar cultivar after 120 h of salt stress and was upregulated at 6 h of salt stress in root tissues of IR29 and Jelodar and shoot tissues of Sang Tarom (Figure 10). It seems that *OsKAT3* is involved in the early rice response to salt stress. In addition, *OsKAT1* was significantly upregulated in response to long-term salt stress (after 120 h of salt stress) in root tissues of all studied rice cultivars, and *OsKAT1* showed an approximately 35-fold increase in expression after 120 h in the Jelodar cultivar. Furthermore, the highest expression of the *OsKAT2* gene was observed in roots after 6 and 24 h of salinity treatment; after 24 h of salinity treatment, this gene showed a 27-fold increase in expression compared to the control (Figure 10). Interestingly, all *OsKAT* genes were upregulated after 6 h of salt stress. Besides, *OsKAT* genes were more highly expressed in root tissues in rice, and their expression levels were repressed in shoot tissues under salt stress. Overall, it seems that the repression of the *OsAKTs*, *OsKAT2*, and *OsKAT2* in roots is related to salinity tolerance in rice.

## 4. Discussion

Potassium (K+) is essential for plant growth and plays important roles in regulating cellular processes such as controlling pH and cell turgor [1,2]; potassium is involved in regulating the activity of many enzymes as an essential cofactor [1,3,4]. K+ channels are involved in the transport and distribution of potassium in plant cells [1,12]. Many *K+ channel* genes have been characterized in different plant species [2,14,15]. In the current study, three *OsKAT* genes and two *OsAKT* genes, along with 11 nonredundant putative potassium channel genes in the rice genome, were characterized based on their physicochemical properties and sequences (Table 1). Potassium channel genes have diverse functions and structures. The exon number varied from 1 to 11, and the pI ranged from 5.78 to 10.39. Diversity in their structures may affect their functions and interaction networks [63]. Regarding the phylogenetic results, a high diversity was observed between potassium channel proteins, and *OsKATs* showed high genetic divergence from other potassium channel proteins. A wide range of *K+ channel* genes have expanded during evolution and obtained diverse functions in plants [12]. A high diversity between potassium channel proteins indicates that the potassium channel gene family originated before the divergence of dicots and monocots [64,65].

The intraspecies synteny results showed that some of the duplicated blocks were collinear, such as *Os01g0210700*, *Os01g0718700,* and *Os01g0756700*, which may demonstrate chromosome segmentation or a large-scale duplication event. Furthermore, the nonsynonymous (Ka) and synonymous (Ks) substitution rates among the duplicated pairs can be considered an important index to assay the selection pressure and approximate duplication time. Because the Ka/Ks ratios were <1 in duplicated gene pairs from the K+ channel family in rice, it can be suggested that the genes underwent purifying selection [66]. These results significantly suggest that the genes with conserved functions and/or pseudogenization might be generated by purifying selection. As to the predicted motifs in K+ channel proteins, it was found that the genes within the duplicated gene group could be functionally conserved. This can be attributed to one or more primeval polyploidy events in multiple angiosperm plant lineages. Therefore, these gene duplications in the rice genome result in the appearance of evolutionary novelties. The syntenic blocks in *K+ channel* genes were investigated across rice and several other plant species, and the closest orthologs of the K+ channels in the rice genome were revealed to be from maize. These wide synteny relations among these species at the gene level were considered to confirm their close evolutionary relationships [67]. Some variations in these evolutionary relationships can illustrate the vast rearrangement events in rice and its related species during the genome evolution process.

Protein structures are precisely associated with the functions of genes and can reflect the phylogenetic relationships among them [64,68]. The protein structures of all the candidate K+ channel proteins were modeled at >90% confidence, and their potential active ligand-binding sites were also identified. Some diversity in these protein structures may reflect their different roles in the transmembrane transport process in response to multiple environments. Based on the three-dimensional structure analysis, it can be mentioned that these proteins from multiple clades belong to functionally diverse groups but share a common catalytic mechanism in the stabilization of membrane potential and cellular potassium ion homeostasis under stresses, supporting that K+ channel proteins can play an important role in intracellular signaling pathways in the response to unfavorable conditions. In protein structures, channels, and cavities are significantly engaged in protein function adjustment and can vary their binding specificities [64,69,70]. This may suggest that K+ channel evolutionary divergence can modulate the characteristics of the gene to function during various molecular pathways. The LEU, VAL, SER, PRO, HIS, GLY, LYS, TYR, CYC, and ARG amino acids were predicted as the binding residues in the ligand-binding site of nearly all candidate K+ channel proteins, which may manifest the importance of these residues in those positions on the DNA molecule and their cellular functional performance. For instance, SER, VAL, PRO, and LEU have been identified as the key amino acids involved in regulating the responses to stress [71,72]. In addition, the potential posttranslational modifications of K+ channel proteins were predicted in terms of N-glycosylation and phosphorylation modifications. Glycosylation and phosphorylation modifications play vital roles in protein functions in eukaryotic organisms [73]. In the current study, OsKAT1 was predicted to be a protein with a high potential for N-glycosylation and phosphorylation modifications. Glycosylation modifications may change the stability and molecular weight of the target protein [73,74]. In addition, phosphorylation modifications play critical roles in protein activity, protein–protein interactions, and the regulation of signal transduction [65,75,76]. Previous studies have revealed that posttranslational modifications such as phosphorylation are involved in activating the K+ channel AKT1 [1,21,31]. Two calcium sensors, calcineurin B-like 1 (CBL1) and CBL2, with a CBL-interacting protein kinase (CIPK 23) are involved in AKT1 phosphorylation [21,77]. Posttranslational modifications may affect the function of K+ channel proteins, especially OsKAT1 and OsAKT1.

The study of co-expression networks reveals valuable information on the possible cellular pathways related to target genes [66]. In the present study, an interaction network of 98 genes c-oexpressed with *K+ channel* genes was constructed in rice. Base on the co-expression network, *OsAKT2* can interact with genes involved in ubiquitin-mediated proteolysis, calcium signaling, and plant–pathogen interactions. Regarding the co-expression network and KEGG ontology results, several metabolic pathways, including amino sugar and nucleotide sugar metabolism, purine metabolism, carbon metabolism, glycerophospholipid metabolism, monoterpenoid biosynthesis, fructose and mannose metabolism, and folate biosynthesis, were recognized in the co-expression network. Regarding these results, K+ channel proteins have critical functions during rice growth and stimuli processing. Due to the importance of potassium in plant growth and development, potassium channels in different tissues have been expanded for the proper distribution of K+ in plant cells [78]. Based on the available RNA-seq data, *K+ channel* genes showed differential expression levels in different tissues in response to biotic and abiotic stresses. For instance, *OsAKT1* is expressed in seeds and roots, while *OsAKT2* is more highly expressed in leaves. It seems that the *OsKAT* and *OsAKT* genes are not specific to an organ/tissue and are expressed as key potassium channels in different tissues of rice. Furthermore, potassium channel genes showed differential expression in response to adverse conditions. As to the expression patterns, *OsAKT1* was upregulated in response to phosphate starvation, while *OsKAT1* was upregulated in response to the rice pathogen *X. oryzae*. In previous studies, the role of *AKTs* and *KATs* in responding to abiotic stresses such as drought, salinity, osmotic stress, and iron toxicity was determined [13,25,26,78]. However, our findings reveal that *OsAKTs* and *OsKATs* are involved in the response to biotic stresses, which shows their importance in the resistance of rice to pathogens.

The relationship between potassium and sodium is a key factor in salt tolerance in plants, and increasing potassium accumulation in the cytoplasm reduces sodium toxicity to organelles [2,13,26]. Regarding the real-time PCR results, *OsAKTs* and *OsKATs* were induced by salt stress in the root and shoot tissues of rice cultivars. *OsAKTs* were more downregulated in response to salt stress, while *OsKATs* were sharply upregulated in root tissues in response to salt stress. In root tissues, *AKT1* can affect Na uptake, and suppression of *AKT1* reduces transpiration in guard cells [79,80]. Another previous study stated that the overexpression of *OsAKT1* might improve rice tolerance to drought stress [81]. It seems that *OsAKT1* downregulation is a part of the response of plants to salinity stress as well as drought stress. *AKT2*, as a potassium channel, contains two different gating modes and is involved in K+ loading and unloading in the phloem [82,83,84]. It was stated that the activity of *AKT2* is suppressed by salt stress [85]. In the current study, *OsKAT3* was identified as an early component of the rice response to salt stress, whereas *OsKAT1* was recognized as a rice response to long-term salt stress in root tissues. However, all *OsKAT* genes were upregulated after 6 h of salt stress. It was reported that *OsKAT1* might increase rice salinity tolerance [26]. In addition, Moon et al. found that *OsKAT2* is involved in drought tolerance in rice by affecting stomatal opening [25]. The function of *OsKAT3* is still unknown, and its role in responding to environmental stresses such as salinity has not been determined. Overall, our results revealed that *OsKAT1* has a high potential for regulating the response of rice to salinity stress. Furthermore, the results of the prediction of posttranslational modifications and co-expression networks indicated that *OsKAT1* is involved in different cellular signaling pathways related to plant tolerance and can be used in future rice molecular breeding programs related to increasing salinity tolerance.

## 5. Conclusions

Potassium channels are key transporters involved in the distribution of potassium in plant cells. In the present study, *OsKAT* and *OsAKT* genes, along with 11 potassium channel members in the rice genome, were widely characterized. Our genomic results showed that potassium channel genes are differentiated in their functions and structures. Furthermore, our bioinformatic analyses revealed new insights into regulatory systems and interaction networks of potassium channel genes in rice. Our findings revealed that repression of *OsAKTs*, *OsKAT2*, and *OsKAT2* in roots is related to salinity tolerance in rice. Moreover, *OsKAT1* is identified as a key gene involved in the rice response to salt stress, and its allelic variants may be used as genetic markers in future rice breeding programs.

## Figures and Tables

**Table 1:** List of studied potassium channel genes in rice.

Gene ID	Description	CDS (bp)	Peptide (aa)	Exon No.	MW (kDa)	pI
*Os01g0648000*	OsAKT1	3389	935	11	104.46	7.21
*Os05g0428700*	OsAKT2, Potassium channel protein ZMK2	1858	566	7	78.54	6.85
*Os02g0245800*	OsKAT1, Inward facing shaker-like potassium channel	2343	718	11	82.97	7.21
*Os01g0210700*	OsKAT2 Potassium channel	2218	568	11	65.85	8.30
*Os01g0756700*	OsKAT3, Shaker potassium channel	1867	502	9	58.01	8.32
*Os01g0696100*	Osrok, Putative outwardly facing K+ channel	1218	405	2	33.54	6.19
*Os06g0254200*	Potassium channel protein NKT5	1833	610	1	67.39	8.98
*Os04g0117500*	Potassium channel, voltage-dependent	1323	286	7	42.79	10.39
*Os12g0118400*	Inwardly facing potassium channel	2053	529	1	58.75	9.18
*Os03g0752300*	TPKA, Two-pore K+ channel family protein	1672	347	3	39.24	8.16
*Os04g0445000*	Potassium channel SKOR (Stelar K(+) outward facing channel)	1646	454	8	81.82	8.48
*Os07g0108800*	TPKB, Two-pore K+ channel family protein	1468	349	1	37.91	6.26
*Os02g0817500*	KOB1, Potassium channel, voltage-dependent, β subunit	1326	328	4	36.45	7.49
*Os09g0299400*	TPK1, Two pore potassium channel c	1513	456	2	49.66	9.24
*Os06g0250600*	OsK5.2, KOR1, Outward facing shaker K+ channel, K+ release by guard cells	3025	858	11	97.38	5.78
*Os01g0718700*	Potassium channel protein	1311	373	7	66.84	9.08

## Supplementary Materials

The following are available online at https://www.mdpi.com/article/10.3390/genes12050784/s1, Table S1: List of designed primers for real-time PCR; Table S2: List of conserved motifs predicted in potassium channel protein sequences of rice; Table S3: The predicted duplicated gene pairs in the potassium channel protein family in rice; Figure S1: Synteny analysis of *K+ channel* genes in rice; Figure S2: The protein channel regions predicted in K+ channel proteins in rice.
