# Supplementary material for: Genome-Wide Analysis of Potassium Channel Genes in Rice: Expression of the OsAKT and OsKAT Genes under Salt Stress
Source: Genes (Basel). 2021 May 20;12(5):784. doi: 10.3390/genes12050784 (PMC8160896; doi:10.3390/genes12050784)
Supplement: Supplementary file 1 [file genes-12-00784-s001.zip › genes-1207119-supplementary.pdf]

genes

MDPI

Supplementary Materials

Genome-wide Analysis of *potassium channel* Genes in Rice: Expression of the *OsAKT* and *OsKAT* Genes under Salt Stress

Zahra Musavizadeh [1], Hamid Najafi-Zarrini [2], Seyed Kamal Kazemitabar [2], Seyed Hamidreza Hashemi [3], Sahar Faraji [2], Gianni Barcaccia [4], Parviz Heidari [5,*]

[1] Department of Biochemical Sciences, Sapienza University of Rome, 00185 Rome, Italy; Zahra.musavizadeh@uniroma1.it (Z.M.)
[2] Department of Plant Breeding, Sari Agricultural Sciences and Natural Resources University (SANRU), Sari 4818166996, Iran; najafi316@yahoo.com (H.N-Z.); kazemi_ka@yahoo.com (S.K.K.); sahar.faraji@rocketmail.com (S.F.)
[3] Genetics and Agricultural Biotechnology Institute of Tabarestan, Sari Agricultural Sciences and Natural Resources University, Sari 4818166996, Iran; irahamidreza@yahoo.com (S.H.H.)
[4] Laboratory of Genomics for Breeding, DAFNAE, Campus of Agripolis, University of Padova, Legnaro, 35020 Padova, Italy; gianni.barcaccia@unipd.it (G.B.)
[5] Faculty of Agriculture, Shahrood University of Technology, 3619995161 Shahrood, Iran
* Correspondence: heidarip@shahroodut.ac.ir; Tel.: +98-912-0734-034

Table S1. List of designed primers for Real-time PCR

Gene	Accession number	Primer (5′-3′)	Gene ID in NCBI	Product size (bp)
OsAKT1	*Os01g0648000*	Forward: TACAGCCGACAATGTGGTGAAT<br>Reverse: GTCAAGAACAAGCCATGTGGTA	LOC4326245	148
OsAKT2	*Os05g0428700*	Forward: CTACCTCATCGGCAACATGAC<br>Reverse: CGAAATTGGAGGCAGCACGAA	LOC4338867	92
OsKAT3	*Os01g0756700*	Forward: CCATGTACTGGTCCATCACGA<br>Reverse: TTGGTCATGTTGCCGATGAGG	LOC4325143	142
OsKAT2	*Os01g0210700*	Forward: GGACCAGGAATTTTAGGGACAC<br>Reverse: CCTTGGGGAGACTATCCAATGT	LOC4325560	159
OsKAT1	*Os02g0245800*	Forward: CCGAGTCAGTTCACTATTTGCC<br>Reverse: TGCGAAGAGGGTCACAGAAATA	LOC4328864	97

Table S2. List of conserved motifs predicted in potassium channel proteins of rice

Motif No.	E-value	Amino acid sequence of motif	Width (aa)
Motif 1	3.6e-273	YWSITTLTTVGYGDLHAENTGEMLFSIAFMLFNLGLTAYJIGNMTNLVVH	50
Motif 2	1.6e-162	RLWRLRRVSKLFARLEKDIRFNYLWTRCIKLJSVTLFAVHCAACI	45
Motif 3	8.8e-139	ZSVYLFKGVSDEFILQLVTEMKAEYFPPKEDIILQNEAPTDVYIVVSGAV	50
Motif 4	8.2e-123	GTSRTRKFRDTIQAASEFGARNQLPEAIKEQMLAHICLRFRTEELLQQEM	50
Motif 5	1.2e-102	VDAFFAIDIALTFFVAYRDKKTYLLVDDPKKIATRYLSSWF	41
Motif 6	4.7e-091	FGEIGVLCNIPQPFTVRTRELCQLLRJSKTALLEIIQENREDGRIILSNL	50
Motif 7	5.8e-071	PSLGATANQSPKLRKFIISPYDPRYRIWETFLIVLVVYSAWICPFELAF	49

Motif 8	8.6e-065	DRYPIPELTWIGAQIPNFEDRSLWFRYVT	29
Motif 9	1.7e-032	AADLDNDGRVGKADFVVYKLKELGKISZEDISMFLDZFDKLDADHSGKJS	50
Motif 10	2.2e-031	WKVEGLGWVDAFYLVVASVTTLGYGDRSFSSPAGRLFAVAWJLVSTJAVA	50
Motif 11	2.9e-029	GADPNESDYDGRTALHIAASKGYEDCVRFLLQQGADVNAKDKFGNTPLWE	50
Motif 12	4.3e-029	LDELPKAIRSSIAQYLFLPVV	21
Motif 13	2.6e-025	LDVASTIPLQIIYQLVTGKRQGL	23
Motif 14	2.1e-016	GFRPTKILTVEGAEIDDIEVIRDGDHLF	28
Motif 15	6.4e-014	YGGDPNARDRDGRTPLHIAAAEGLYLVAKLLVENGADIDAKDRWGNTP	48

Table S3. The predicted duplicated gene pairs in the potassium channel protein family in rice

Gene 1	Gene 2	Ka	Ks	Ka/Ks	p-value	Divergence time (MYA)
Os01g0756700	*Os01g0210700*	0.26	1.1	0.236363636	1.04E-06	84.61538462
Os01g0210700	*Os02g0245800*	0.29	1.53	0.189542484	2.52E-08	117.6923077
Os03g0752300	*Os07g0108800*	0.28	2.66	0.105263158	1.30E-06	204.6153846
Os06g0254200	*Os04g0117500*	0.31	1.42	0.218309859	3.28E-05	109.2307692
Os06g0254200	*Os01g0718700*	0.35	1.83	0.191256831	1.46E-08	140.7692308

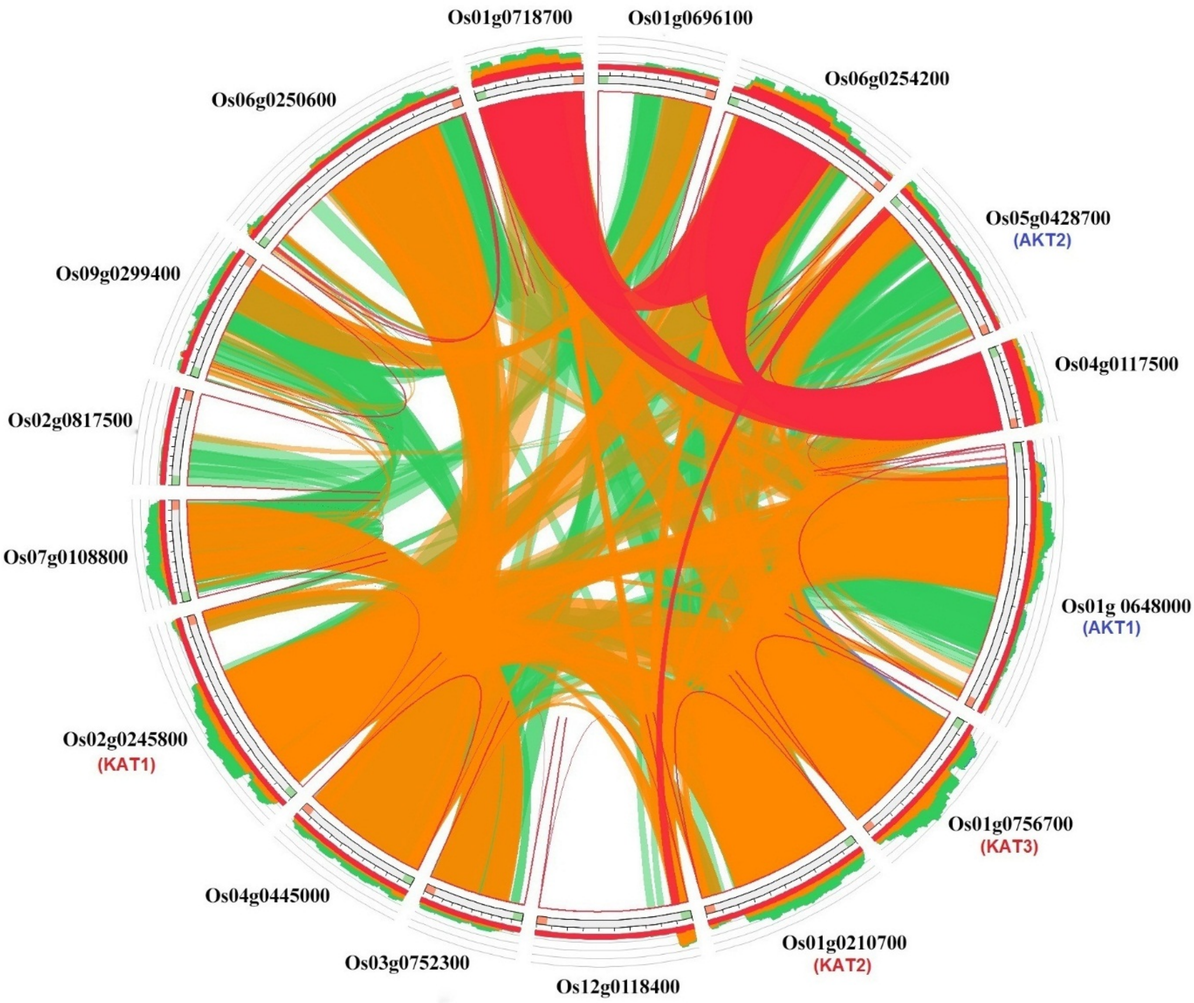

Figure S1. Synteny analysis of *K+ channel* genes in rice

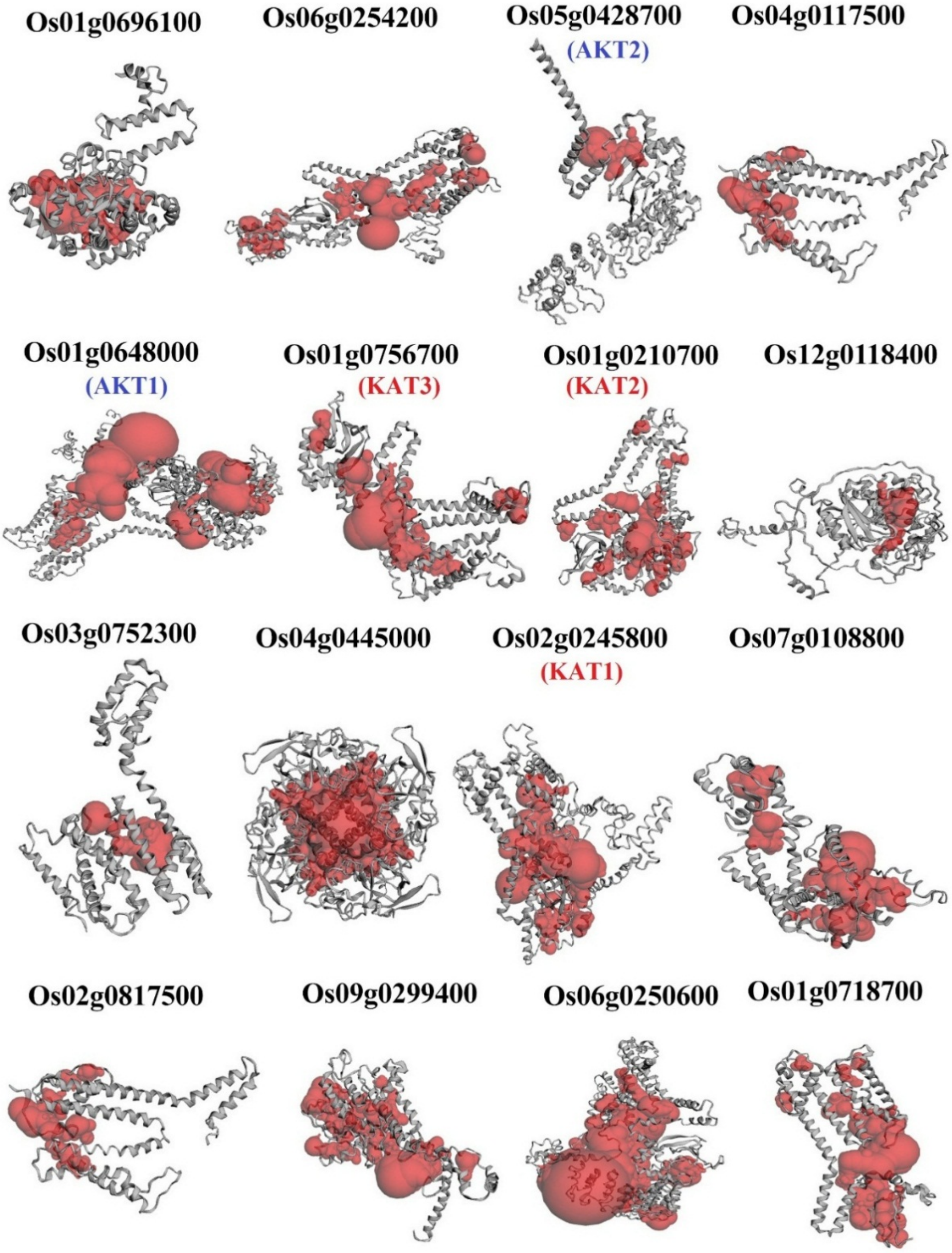

Figure S2. The protein channel regions predicted in K+ channel proteins in rice
